# Breaking point: Systemic mastocytosis manifesting as severe osteoporosis

**DOI:** 10.18632/oncoscience.614

**Published:** 2025-02-04

**Authors:** Areti Kalfoutzou, Kalliroi Spanou, Adam Mylonakis, Vassiliki Lagopoulou, Maria Dimitrakoudi, Alexandra Korovila, Christos Piperis, Eleni Tsiouri, Eleni Mostratou

**Affiliations:** ^1^Department of Medical Oncology, 251 Air Force General Hospital, Athens 115 25, Greece; ^2^Department of Pathology, 251 Air Force General Hospital, Athens 115 25, Greece; ^3^First Department of Surgery, “Laiko” General Hospital, National and Kapodistrian University of Athens 115 27, Greece; ^4^Department of Hematology, “Laiko” General Hospital, National and Kapodistrian University of Athens 115 27, Greece; ^5^Department of Radiation Oncology, Alexandra Regional General Hospital, Athens 115 28, Greece; ^6^Department of Cardiology, “Georgios Gennimatas” General Hospital, Athens 115 27, Greece; ^7^Second Department of Internal Medicine, 251 Air Force General Hospital, Athens 115 25, Greece

**Keywords:** mastocytosis, mast cells, osteoporosis, anaphylaxis

## Abstract

Systemic mastocytosis (SM) encompasses a wide spectrum of myeloproliferative disorders defined by the aggregation of abnormal mast cells in various tissues, including the bone marrow, gastrointestinal tract, liver and lymph nodes. The release of tryptase, interleukins and cytokines by the accumulated mast cells causes a multi-system response that can range from mild flushing and pruritus to severe anaphylactic reactions, gastrointestinal disturbances, and cardiovascular symptoms, including hypotension and syncope. Furthermore, severe osteoporosis manifesting as bone-lytic lesions or pathologic fractures due to mast cell mediator-triggered bone resorption, is a rather common manifestation of SM, occurring in more than two-thirds of patients. The vast majority of SM cases harbor the D816V KIT mutation, which is an independent prognostic factor, and serves as a therapeutic target. This is a rare case of a young male who presented with new-onset back pain due to osteoporotic fractures and was diagnosed with SM without the D816V KIT mutation. Our case aims to emphasize one of the most underrecognised causes of osteoporosis in adults, and to shed light on a frequently misdiagnosed yet potentially severe hematologic disorder.

## INTRODUCTION

SM is a rare myeloproliferative neoplasm with an incidence of 1 per 10.000 people worldwide [1]. It arises from the clonal proliferation of abnormal mast cells, driven primarily by mutations in the KIT oncogene, with the D816V KIT mutation being the most frequent [2]. The rarity of SM, combined with its highly variable presentation, often leads to significant diagnostic delays.

As opposed to cutaneous mastocytosis (CM), which is encountered mostly in pediatric populations, SM typically occurs in middle-aged adults and is characterized by multi-organ involvement and a diverse clinical spectrum [3]. Symptoms such as osteoporosis, gastrointestinal disturbances, or flushing, induced by mast cell activity and tissue infiltration, overlap with more common conditions like primary osteoporosis or irritable bowel syndrome, causing SM to go undiagnosed for several years [4]. Bone involvement with osteoporosis, skeletal pain, and pathologic fractures is common in SM, occurring in up to 50% of patients, and is primarily attributed to mast cell mediator-induced bone resorption and infiltration of the bone marrow [1]. Interestingly, 2–19% of SM cases present with osteosclerotic lesions and increased bone density, which are associated with a poorer prognosis [1].

Our patient, who had a history of multiple sclerosis (MS), presented with sudden-onset back pain in the absence of trauma. Imaging revealed pathologic vertebral fractures with diffuse osteoporotic changes throughout the spine. Subsequent evaluation demonstrated elevated serum tryptase levels and abnormal mast cell aggregates infiltrating the bone marrow, confirming the diagnosis of SM.

This report highlights the diagnostic challenges of SM, emphasize its underrecognized role in osteoporosis and fragility fractures, and advocates for its consideration in patients with unexplained skeletal abnormalities. By presenting this rare case, we aim to contribute to the understanding of SM, its molecular variations, and its rare coexistence with other chronic conditions, including MS, which share overlapping symptoms.

## CASE PRESENTATION

A 23-year-old male was referred to our department due to worsening lower back pain and difficulty walking over the past month. He did not report any recent fall or accident. His past medical history was significant for multiple sclerosis (MS) under treatment with ozanimod and glucose-6-phosphate dehydrogenase deficiency (G6PD) deficiency. Neurological examination revealed a patient well-oriented in space and time, with reduced lower limb mobility due to lumbar pain, no focal neurological deficits, and bilateral positive Laseque signs. No lymphadenopathy or hepatosplenomegaly was observed.

**Figure 1:**
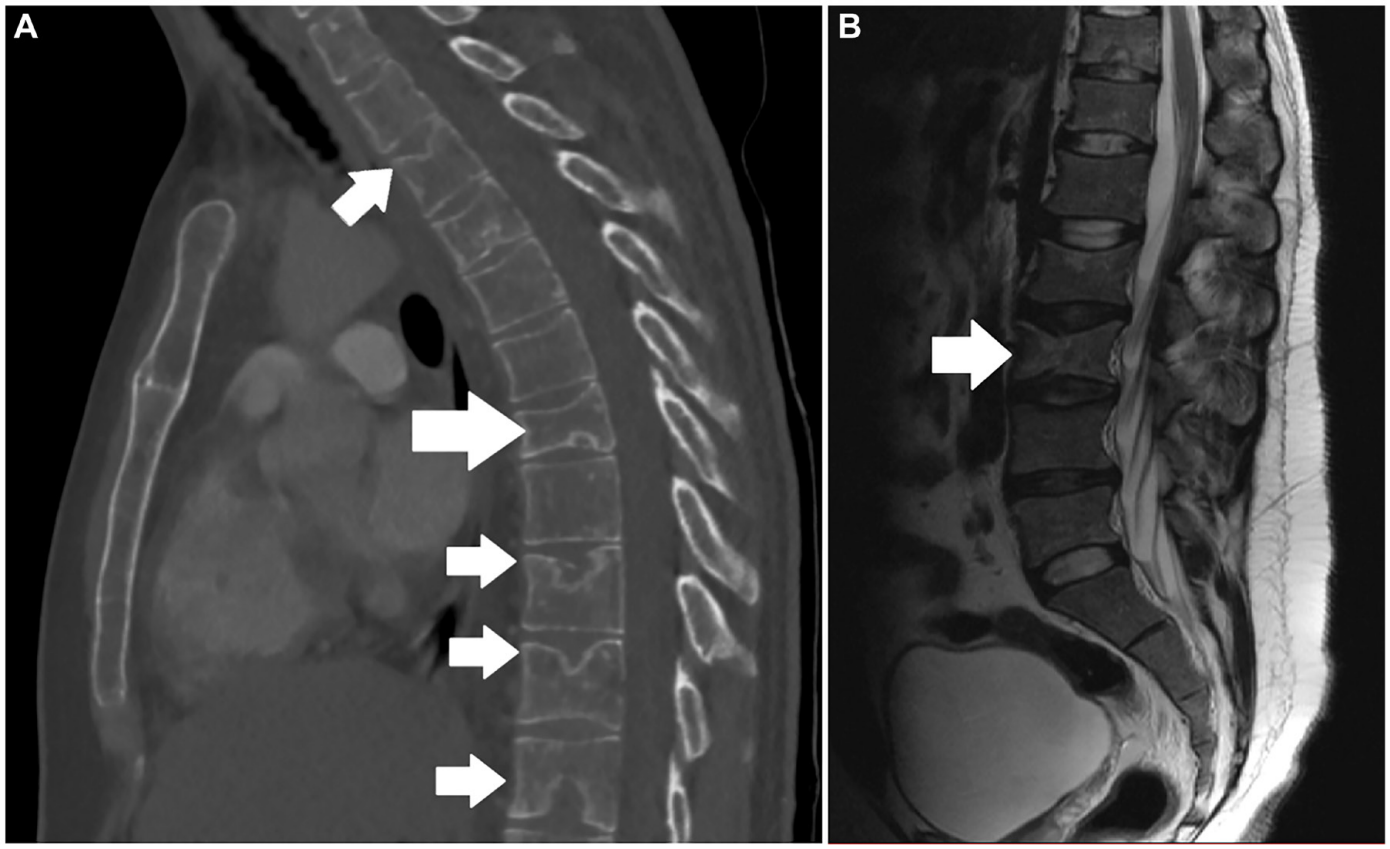
(**A**) Sagittal CT scan of the chest demonstrating a compression fracture in the T7 vertebra (large white arrow), along with osteoporotic changes in the epiphyseal plates of multiple vertebrae (smaller white arrows). (**B**) Sagittal MRI scan of the lumbar spine revealing a compression fracture in L3 (white arrow).

Laboratory examinations were unremarkable, apart from normochromic normocytic anemia and a slightly elevated white blood cell (WBC) count with a neutrophilic predominance (Table 1). A computed tomography (CT) scan of the chest demonstrated a wedge-shaped T7 fracture, along with osteoporotic changes in the epiphyseal plates of several vertebrae (Figure 1A). Additionally, an MRI scan with intravenous contrast revealed similar findings throughout the lumbar spine, along with an L3 vertebral fracture (Figure 1B). A Dual-energy X-ray Absorptiometry (DEXA) scan indicated severe osteoporosis (T-score:-3.3) in the lumbar spine.

**Table 1:** Laboratory values of our patient upon admission

Laboratory examination	Patient’s values	Reference range
White Blood Cells	13.5	4–10 × 10^9^/L
Neutrophils	12.7	1.5–7 × 10^9^/L
Hemoglobin	131	140–180 g/L
Hematocrit	41.2	41–50%
MCV	98	79–98 fl
MCHC	328	320–360 g/d0L
Platelets	384	14–440 × 10^9^/L
C-Reactive Protein	3.25	0–10 mg/L
Glucose	6.11	3.88–6.11 mmol/L
BUN	9.28	5.3–19.2 mmol/L
Creatinine	106	61.8–114.9 µmol/L
AST	25	5–40 U/L
ALT	39	5–45 U/L
ALP	79	35–116 U/L
Albumin	4	3.5–5.5 g/L
Ferritin	165	10–291 μg/L
B12	121	57–247 pmol/L
Folate acid	6.63	>1.69 nmol/L
K	4.2	3.5–5.3 mmol/L
Na	138	137–150 mmol/L
Ca	2.3	2–2.6 mmol/L
P	0.13	0.13–0.27 mmol/L	

Abbreviations: MCV: Mean Corpuscular Volume; MCHC: Mean Corpuscular Hemoglobin Concentration; BUN: Blood Urea Nitrogen; AST: Aspartate Aminotransferase; ALT: Alanine Aminotransferase; ALP: Alkaline Phosphatase.

**Figure 2:**
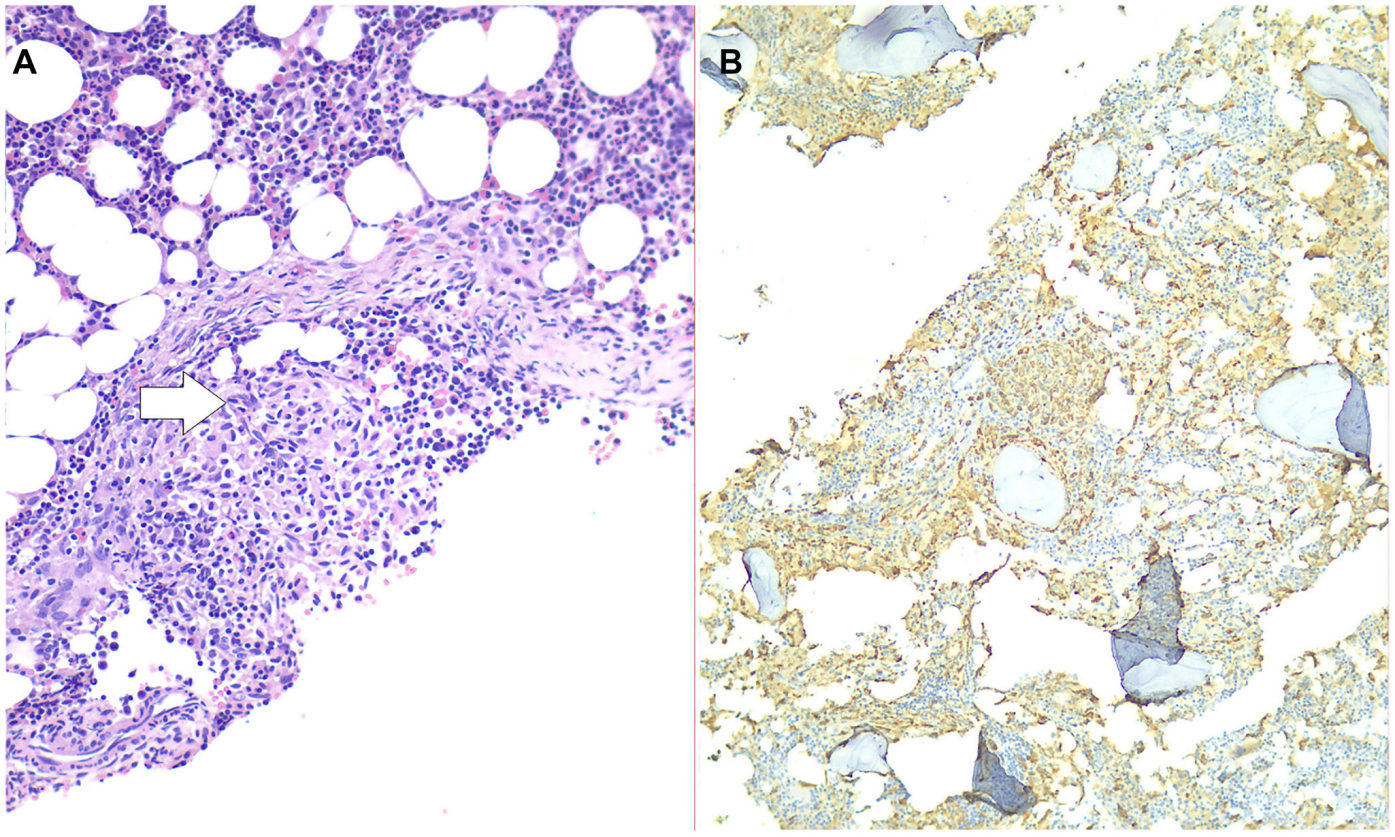
(**A**) Hematoxylin - eosin stain, 20x magnification: Histopathological examination demonstrating mast cell aggregates infiltrating the bone marrow (white arrow). (**B**) Immunohistochemically, the cells stain positive for tryptase (10x magnification).

**Figure 3:**
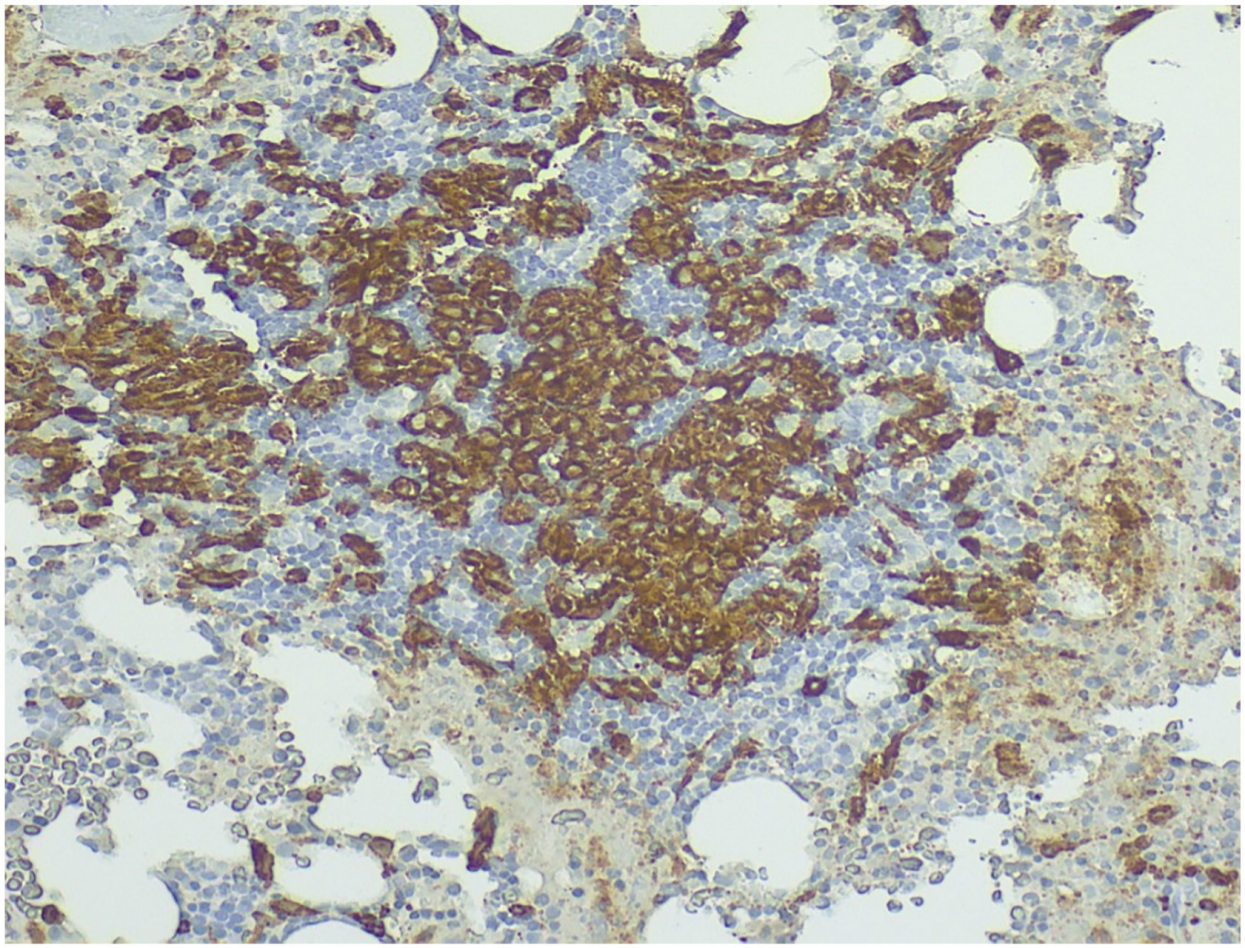
Immunohistochemical staining showing CD117 (KIT) positivity in mast cells (20x magnification).

To exclude causes of secondary osteoporosis, a comprehensive laboratory work-up was performed, including measurement of serum calcium, phosphate, parathyroid hormone (PTH), 25-hydroxyvitamin D, thyroid-stimulating hormone (TSH), and cortisol levels, all of which were within normal limits (Table 2). Elevated serum tryptase and C-terminal telopeptide of collagen (CTx) levels were also observed, prompting a bone marrow biopsy. Histopathology revealed 15–20% bone marrow infiltration by multifocal aggregates of abnormal, spindle-shaped mast cells (Figure 2A). The mast cells stained positive for tryptase (Figure 2B), CD117 (Figure 3) and CD30, and negative for CD123, CD2 and CD25. Molecular testing revealed the absence of the KIT D816V mutation. Α subsequent full-panel molecular analysis for other KIT mutations was recommended, but the patient declined.

**Table 2:** Osteoporosis laboratory work-up of our patient

Laboratory examination	Patient’s values	Reference range
24-hour urine Calcium	2.825	2.5–7.5 mmol/24 h
Parathormone	4.5	0–6.5 pmol/L
25(OH)D	82	62.5–200 nmol/L
ACTH	8.5	<8.8 nmol/L
Serum free cortisol	17.2	3.7–19.4 μg/L
Free serum testosterone	0.62	0.174–0.867 nmol/L
Serum tryptase	28.5	<11.4 μg/L
BAP	13.4	5.5–22.9 μg/L
PINP	41.4	22–85 μg/L
CTx	0.8	0.06–0.7 μg/L	

Abbreviations: 25(OH)D: 25-hydroxyvitamin D; ACTH: Adrenocorticotropic Hormone; BAP: Bone Alkaline Phosphatase; PINP: Amino-terminal propeptide of type I procollagen; CTx: C-terminal telopeptide of collagen.

The coexistence of SM, MS, and G6PD deficiency in this patient presents a unique diagnostic and therapeutic challenge. While MS or its treatment with ozanimod could theoretically exacerbate SM-related immune dysregulation, no clear evidence exists to support such an interaction. G6PD deficiency, however, necessitated careful selection of NSAIDs for pain management to avoid hemolysis. In this case, ibuprofen was chosen due to its safety profile in G6PD deficiency.

Based on the World Health Organisation (WHO) 2022 diagnostic criteria, our patient met 1 major and 2 minor criteria for the diagnosis of SM with bone marrow involvement (Table 3) [5]. Zoledronic acid at a dose of 4 mg was administered to treat osteoporosis and reduce the risk of further fractures, as supported by studies demonstrating its efficacy in such cases [6]. A thoracolumbar spinal brace was applied for stabilization of the fractures. The patient was discharged after 10 days on nonsteroidal anti-inflammatory drugs (NSAID), auto-injectable epinephrin pens, prophylactic anticoagulation, as well as oral vitamin D and calcium supplementation. He was referred to a specialized center for the initiation of systemic treatment with avapritinib, given the extensive bone marrow and skeletal involvement, the KIT 816V-negative status and the CD30 expression, which suggested an aggressive disease phenotype. He has not experienced another bone fracture is scheduled for a follow-up DEXA scan 3 months post-discharge to assess the initial response to treatment and monitor bone density changes. Long-term follow-up of his bone mineral density and hematological parameters is planned to ensure sustained improvement and early detection of any complications. Written informed consent was obtained from the patient for the publication of this case report and any accompanying images.

**Table 3:** The WHO 2022 Diagnostic criteria for SM

Diagnostic criteria
**Major criteria**	Multifocal dense aggregates of mast cells on bone marrow or other extracutaneous organ tissue biopsy
**Minor criteria**	>25% mast cells with atypical morphology Presence of D816V or other KIT activating mutation Serum total tryptase > 20 ng/mL Mast cells with CD2, CD25, and/or CD30 expression

1 major + 1 minor or at least 3 minor criteria are required for establishing diagnosis.

## DISCUSSION

Mastocytosis refers to a wide range of hematologic disorders characterised by the clonal proliferation and accumulation of abnormal mast cells in the skin (cutaneous mastocytosis – CM) as well as extracutaneous organs and tissues (systemic mastocytosis – SM), including the bone marrow, gastrointestinal (GI tract), liver and spleen [7–9]. SM accounts for 10% of mastocytosis and occurs most commonly in middle-aged adults with a slight male predominance [10–12]. According to the WHO 5th edition classification, it is categorized as indolent SM (ISM), bone marrow mastocytosis, smoldering SM (SSM), SM with an associated hematologic neoplasm (SM—AHN), aggressive SM (ASM) and mast cell leukemia (MCL) [3, 9].

The pathogenesis of systemic mastocytosis (SM) is driven by the clonal proliferation of abnormal mast cells, primarily due to an oncogene driver mutation and the subsequent release of inflammatory mediators such as histamine and prostaglandin D2 [7]. A gain-of-function mutation in the KIT proto-oncogene, identified in more than 80% of SM patients, plays a key role in the maturation, proliferation and survival advantage of the mast cells [13, 14]. The most common mutation, accounting for more than 90% of KIT mutations in SM, is the D816V mutation, resulting to the substitution of aspartic acid to valine at the 816 codon [4, 12]. Less common KIT mutations, as well as mutations in the ASXL1, RUNX1, TET2, SRSF2, JAK2 or N/KRAS genes are also implicated in SM pathogenesis and significantly affect patient prognosis [15].

The clinical course varies from asymptomatic to highly aggressive, depending on the subtype and extent of mast cell infiltration on tissues. Anaphylactic symptoms such as urticaria, pruritus, flushing and angioedema are quite common, whereas more severe reactions present with hypotension, chest pain or syncope [2, 8, 16, 17]. Gastrointestinal manifestations, occurring in approximately 60% of patients, include abdominal pain, nausea/vomiting, malabsorption and diarrhea, often requiring a gastrointestinal (GI) endoscopy with biopsies for diagnosis [10, 12, 18]. Mast cell infiltration of the liver results in hypoalbuminemia, ascites or portal hypertension [7]. Additionally, bone involvement is encountered in up to 50% of cases, leading to osteoporotic fractures and skeletal pain [1]. Our case presented with difficulty walking, skeletal pain and multiple vertebral fractures.

Anemia, leucopenia or thrombopenia suggest extensive bone marrow infiltration, whereas some cases exhibit eosinophilia [17, 19]. Biochemical tests demonstrate nutrient deficiency due to malabsorption in severe gastrointestinal disease, or signs of hepatic disfunction in cases with liver infiltration [16, 19]. Elevated serum tryptase is a common finding, and several patients exhibit disturbed markers of bone resorption, such as an elevated C-terminal telopeptide of collagen (CTx) [1, 20]. Imaging studies reveal osteoporotic fractures or focal osteosclerosis, hepatosplenomegaly, or lymph node enlargement depending on the organ involved [8]. The diagnostic complexity of SM-related osteoporosis is further highlighted in the scoping review by Mauro et al. [21], which highlights the correlation between elevated serum tryptase levels, mast cell infiltration in bone marrow, and bone remodeling markers such as C-terminal telopeptide of collagen (CTx). These findings were critical in our case, where elevated serum tryptase and CTx were pivotal in identifying SM as the underlying cause of the patient’s severe osteoporosis. Our patient exhibited normochromic normocytic anemia, likely due to bone marrow involvement, alongside elevated serum tryptase and CTx levels, indicating a systemic disorder involving both mast cell activation and abnormal bone remodeling. Imaging scans further revealed multiple vertebral fractures and several osteoporotic lesions throughout the spine, reflecting advanced osteoporosis.

Differential diagnosis of SM-related osteoporosis requires a comprehensive laboratory and imaging work-up and includes disorders of bone remodeling, such as hyperthyroidism, primary hyperparathyroidism, Cushing syndrome, glucocorticoid-induced osteoporosis, Paget’s disease, chronic kidney disease (CKD), familial hypocalciuric hypercalcemia, lymphoma and multiple myeloma, or bone metastases, among others [11]. Definitive diagnosis relies on visualization of multifocal mast cell aggregates in a bone marrow or extracutaneous tissue biopsy specimen [5, 12, 19]. Immunohistochemically, positive staining for tryptase and CD117 (c-kit) confirm mast cell lineage, while CD25, CD30 and CD2 distinguish SM from reactive mastocytosis or other myeloid neoplasms [19]. Particularly the CD30 expression is rather unique to aggressive forms of SM [22]. The expression of these markers, which is highly specific for SM diagnosis can be identified through immunohistochemistry or flow cytometry, and is currently implemented in the WHO 2022 diagnostic criteria for SM [5, 19].

The WHO 2022 diagnostic criteria provide a reliable aid, requiring the presence of one major and one minor, or at least three minor criteria to establish diagnosis (Table 3) [5]. Our case met one major (aggregates of abnormal mast cells on bone marrow biopsy) and 2 minor criteria (CD30 expression on mast cells and serum tryptase >20 ng/mL). The patient’s presentation aligns with bone marrow mastocytosis (a recognized subtype under the WHO 5th edition classification), characterized by multifocal mast cell infiltration in the bone marrow without skin lesions [5].

The clinical course is benign in most indolent SM cases but requires a close-follow up, due to a 1–5% risk of progression to a more aggressive subtype, which carries a life expectancy of about 1 to 5 years, depending on the associated myeloid disorder [3]. Treatment in indolent cases focuses mainly on symptom control with H1 and H2 antagonists, proton pump inhibitors, corticosteroids or anti-IgE antagonists such as omalizumab [4]. Patients are strongly advised to avoid potential anaphylactic triggers such as alcohol, specific foods or drugs, and carry self-injectable epinephrine pens [9].

The role of denosumab and bisphosphonates in the treatment of advanced osteoporosis associated with SM is also strongly supported in current literature [1, 21]. The scoping review by Mauro et al. [21] reinforces the efficacy of bisphosphonate therapy, particularly zoledronic acid, in improving bone mineral density (BMD) and reducing fracture risk in SM-related osteoporosis. Additionally, it highlights denosumab as a viable alternative, especially in patients intolerant to bisphosphonates, and its role in lowering serum tryptase levels, reflecting a reduction in mast cell burden [21]. This aligns with the treatment strategy in our case, where zoledronic acid was chosen as the initial therapy to manage aggressive osteoporosis and prevent further fractures.

For more aggressive forms of SM, cyto-reductive therapies such as cladribine and interferon-a, along with multi-tyrosine kinase inhibitors (TKIs) like midostaurin (with or without prednisone) or avapritinib, have proven effective in SM regardless of mutational status [23]. The KIT D816V mutation, which is encountered in over 90% of SM cases, confers resistance to standard TKIs such as nilotinib, dasatinib and imatinib [23]. Bezuclastinib, a novel oral TKI targeting is currently being investigated for KIT 816V positive SM [24]. In aggressive forms of KIT D816V-negative SM or cases with unknown mutational status, TKIs including imatinib, midostaurin or avapritinib are effective options [23]. Notably, avapritinib has demonstrated efficacy in non-KIT gene mutations, including ASXL1 and RUNX1, which are typically associated with advanced disease subtypes and poorer outcomes [23]. Allogeneic hematopoietic cell transplant (HCT) is an option for select cases of relapsed ASM or SM-AHN, whereas the role of CAR-T cells remains to be elucidated [13]. In our case, avapritinib was chosen over imatinib or midostaurin due to its higher reported efficacy in KIT-mutation negative cases [24].

To our knowledge, this is the 8th case of mastocytosis with coexisting MS reported in literature [25]. The patient’s symptoms could have been attributed to MS, as it can also cause low back pain or difficulty walking; however, further investigation was warranted. Potential drug-drug interactions between avapritinib and ozanimod posed a significant challenge, as both drugs are metabolized by the cytochrome P450 enzymes [26, 27]. While no significant contraindications are documented, caution is advised, as potential drug interactions could potentially alter therapeutic efficacy or increase toxicity. Patient’s history of G-6PD deficiency further complicated the case by limiting our choice of anti-inflammatory drugs for pain management [28].

## CONCLUSIONS

SM should be considered in any patient with unexplained osteoporosis, chronic gastrointestinal symptoms or idiopathic anaphylaxis. Particularly in cases of osteoporotic fractures in an otherwise healthy adult, an extensive workup including serum tryptase measurement and a bone marrow biopsy should be carried out in order to rule out SM, which is highly manageable if detected at an early stage. Patients suspected of having SM should be promptly referred to specialized centers for comprehensive follow-up, expert treatment, and facilitated access to clinical trials. Further studies are needed to explore KIT 816SM without the D816V mutation, due to its distinct clinical characteristics and variable treatment responses.
